# Thermal Cycling Effect on Transformation Temperatures of Different Transformation Sequences in TiNi-Based Shape Memory Alloys

**DOI:** 10.3390/ma12162512

**Published:** 2019-08-07

**Authors:** Shyi-Kaan Wu, Yi-Ching Chang

**Affiliations:** 1 Department of Materials Science and Engineering, National Taiwan University, Taipei 106, Taiwan; 2 Department of Mechanical Engineering, National Taiwan University, Taipei 106, Taiwan

**Keywords:** TiNi-based shape memory alloys, thermal cycling, martensitic transformation sequence, transformation temperature, shear strain

## Abstract

In TiNi-based shape memory alloys (SMAs), the effects of thermal cycling on the transformation peak temperatures of B2 ↔ B19′, B2 ↔ R, B2 ↔ B19, B2 ↔ R ↔ B19′, and B2 ↔ B19 ↔ B19′ one-stage and two-stage transformations have been investigated and compared. Experimental results of the differential scanning calorimeter and hardness tests indicate that the alloy’s intrinsic hardness and the shear strain, ***s***, associated with martensitic transformation, are two important factors, due to their relation to the ease of introducing dislocations during cycling. The temperature decrease by cycling for one-stage transformation was in the order of B2 ↔ B19′ > B2 ↔ B19 > B2 ↔ R according to the orders of magnitude of their ***s*** values. This phenomenon also affected the suppression of B19 ↔ B19′ and R ↔ B19′ transformation peak temperatures in two-stage transformation. Both Ti_50_Ni_48_Fe_2_ and Ti_48.7_Ni_51.3_ SMAs aged at 450 °C for 4 h exhibited B2 ↔ R ↔ B19′ transformation, but the hardness of the latter was much higher than that of the former due to the precipitation hardening of the Ti_3_Ni_4_ precipitates. This causesd the decrease of the R ↔ B19′ transformation peak temperature in the Ti_50_Ni_48_Fe_2_ SMA to be much higher than that in Ti_48.7_Ni_51.3_ SMAs aged at 450 °C for 4 h, which directly affected the sequential B2 ↔ R transformation of Ti_50_Ni_48_Fe_2_ SMA in the next thermal cycle and decreased this transformation peak temperature. The Ti_48_Ni_52_ SMA aged at 600 °C for 150 h underwent B2 ↔ B19′ transformation and then B2 → R → B19′/B19′ → B2 transformation as the cycle number increased, in which the B2 ↔ R transformation peak temperature raised slightly by cycling. This characteristic is uncommon and may have resulted from the strain field around the thermal-cycled dislocations favoring the formation of the R-phase.

## 1. Introduction

TiNi-based shape memory alloys (SMAs) are the most well-known SMAs due to their superior shape memory effect (SME), pseudoelasticity (PE), and damping capacity [1,2,3]. Equiatomic TiNi SMA exhibits thermoelastic martensitic transformation, which is associated with the transformation sequence of the B2 parent phase ↔ B19′ monoclinic martensite [1,2]. Due to the formation of Ti_3_Ni_4_ precipitates (ppts), which have a coherent interface with the matrix, solution-treated and low temperature aged Ni-rich TiNi-based SMAs undergo the transformation sequences of B2 ↔ R-phase ↔ B19′ martensite (or B2 → R-phase → B19′ in cooling/B19′ → B2 in heating), and they can only exhibit B2 ↔ R-phase transformation if R-phase ↔ B19′ transformation is suppressed to below the measurable temperature [1,2]. Here, the R-phase is a premartensite with a rhombohedral structure. Altering the composition of Ti_50_Ni_50_ binary SMA to Ti_50_Ni_50-x_A_x_ ternary SMAs, where A is a third element substituted for Ni, also changes the martensitic transformation sequence. For example, in ternary Ti_50_Ni_50-x_Fe_x_ SMAs with higher Fe content, the B2 → B19′ transformation is suppressed and the occurrence of the premartensite R-phase is enhanced [4,5]; i.e., the transformation sequence changes from B2 ↔ B19′ to B2 ↔ R-phase ↔ B19′ and then to B2 ↔ R-phase. Another example is ternary Ti_50_Ni_50-x_Cu_x_ SMAs. The transformation sequence of Ti_50_Ni_50-x_Cu_x_ SMAs changes from B2 ↔ B19′ with a Cu content <7.5%, to B2 ↔ B19 ↔B19′ with a Cu content of 7.5% to 12.5%, and then to B2 ↔ B19 with a Cu content ≥15% [6,7]. Here, the B19 phase is another premartensite with an orthorhombic structure. Similar transformation sequences also occur in other ternary alloys, where A is Au, Pd, etc. [8,9,10]. The transformation sequence of solution-treated Ti_50-y_Ni_50_B_y_ ternary SMAs, where B is Zr and Hf substituted for Ti, is B2 ↔ B19′. These SMAs have higher transformation temperatures than that of Ti_50_Ni_50_ SMA, and are recognized as high temperature SMAs [11,12].

The stability of the transformation temperatures of SMAs is important during engineering applications of SME and PE, because the characteristics of SME and PE are altered if the transformation temperatures change or the transformation temperature range from transformation starting to finishing temperatures increases. It is well-known that the thermal cycling conducted on TiNi-based SMAs will affect their martensitic transformation temperatures, and thus change their SME/PE performance, for example, in SMAs used in mini actuators and micro-electromechanical systems (MEMS) [13,14], applications of elastocaloric effect associated with the martensitic transformation in SMAs [15], etc. In 1986, Miyazaki et al. investigated the effect of thermal cycling on the transformation temperatures of solution-treated and quenched Ti_50.2_Ni_49.8_, Ti_49.4_Ni_50.6_ and Ti_48.4_Ni_51.6_ (all in at. %) SMAs [16]. They found that the transformation temperatures associated with B2 ↔ B19′ were decreased by thermal cycling, irrespective of the Ni content, and that the temperature changes were rapid during the initial cycling but became more gradual later. From transmission electron microscope (TEM) observations, the decrease of the transformation temperature of the SMAs was revealed by the introduction of dislocations during thermal cycling. Such dislocations have a <010>_B2_ Burgers vector, which will not form any antiphase boundary and thus does not decrease the degree of order in the TiNi-based SMAs. In 1994, Liu et al. demonstrated from thermodynamic analysis of the martensitic transformation in Ti_49.8_Ni_50.2_ (in at. %) SMA that the main effect of thermal cycling on the decrease of transformation temperatures under zero stress is due to the increase of the alloy’s elastic strain energy associated with transformation-induced elastic stresses caused by the presence of defects and the internal stress field of the transformation [17,18]. It was shown in Reference [16] that the transformation temperatures were detected by the electric resistivity method instead of differential scanning calorimeter (DSC) measurement. In DSC measurement, there is a transformation peak associated with each martensitic transformation of TiNi-based SMAs. The effect of thermal cycling on the transformation peak temperature can be more clearly identified by DSC results than by electric resistivity curves, so, recently, DSC measurement has become frequently used to measure the thermal cycling effect on the transformation temperatures of TiNi-based SMAs [19,20,21,22,23]. However, to the best of our knowledge, the effects of thermal cycling on TiNi-based SMAs with different transformation sequences have not been systematically investigated or compared. In this study, TiNi-based SMAs with different compositions but the same transformation sequence were selected to investigate the effects of thermal cycling on their transformation temperatures with DSC measurement. Five different transformation sequences were exhibited in TiNi-based SMAs, including B2 ↔ B19′ in Ti_50_Ni_50_ SMA and 600 °C × 150 h-aged Ti_48_Ni_52_ SMA with cycle number less than 20, B2 ↔ R-phase in Ti_50_Ni_46_Fe_4_ SMA and 350 °C × 24 h-aged Ti_48.7_Ni_51.3_ SMA, B2 ↔ B19 in Ti_50_Ni_35_Cu_15_ SMA and Ti_50_Ni_37_Pd_13_ SMA, B2 ↔ R-phase ↔ B19′ in Ti_50_Ni_48_Fe_2_ SMA and 450 °C × 4 h-aged Ti_48.7_Ni_51.3_ SMA, and B2 ↔ B19 ↔ B19′ in Ti_50_Ni_40_Cu_10_ SMA. The causes of the different thermal cycling effects on different transformation sequences have also been discussed. From the viewpoint of the stability of the transformation temperature affected by the thermal cycling, the results of this study can provide the best choice for the SMA’s composition/transformation sequence for SME/PE applications.

## 2. Experimental Procedures

The binary Ti_50_Ni_50_, Ti_48.7_Ni_51.3_, and Ti_48_Ni_52_ SMAs and the ternary Ti_50_Ni_48_Fe_2_, Ti_50_Ni_46_Fe_4_, Ti_50_Ni_35_Cu_15_, Ti_50_Ni_37_Pd_13_, and Ti_50_Ni_40_Cu_10_ SMAs were selected for this study. The SMAs were fabricated from raw materials of titanium, nickel, and other metals (all of purity ≥ 99.9 wt. %) with six cycles of remelting in a vacuum arc remelter (VAR), in which a pure titanium block was used as a getter. The weight loss during the remelting was less than 1 × 10^−5^. The as-melted ingot was hot-rolled at 900 °C into a plate with a thickness of about 2 mm, and then solution-treated at 900 °C for 1 h and quenched in ice water. The oxidation layer of the plate was chemically etched by a solution composed of HF:HNO_3_:H_2_O = 1:5:20 (in volume ratio) and then polished with sandpaper. The solution-treated and quenched plate was cut with a diamond saw into small DSC specimens with weights of ≤50 mg. These have been referred to as the as solution-treated specimens in this study. The as solution-treated specimens of Ti_48.7_Ni_51.3_ and Ti_48_Ni_52_ (in at. %) SMAs were further sealed into evacuated quartz tubes and aged at 350 °C × 24 h or 450 °C × 4 h for the former, and at 600 °C × 150 h for the latter, before being quenched in water. The transformation temperatures of the specimens were determined by a DSC with TA 25 equipment (TA Instruments, New Castle, DE, USA). The thermal cycling test was conducted in situ in DSC equipment, with cycling numbers N of 1 to 50, in which the transformation peak temperatures were identified. For each thermal cycling test, the testing temperatures were set between T_max_ and T_min_, where the martensitic transformation temperature(s) occurred. During the cyclic test, the specimen was held at the T_max_ and T_min_ temperatures for 1 min and run at a constant temperature rate of 10 °C/min between them. The microhardness of the specimen was determined at room temperature (RT) using an Akashi MVK-E Vickers tester (Mitutoyo Corp., Sakado, Kanagawa, Japan) with a load of 4.9 N applied for 15 s. Ten tests were performed on each specimen, and the average Vickers microhardness value of each specimen was calculated from eight tests with the largest and the smallest values excluded.

## 3. Results

### 3.1. B2 ↔ B19′ One-Stage Transformation Sequence

**Figure 1:**
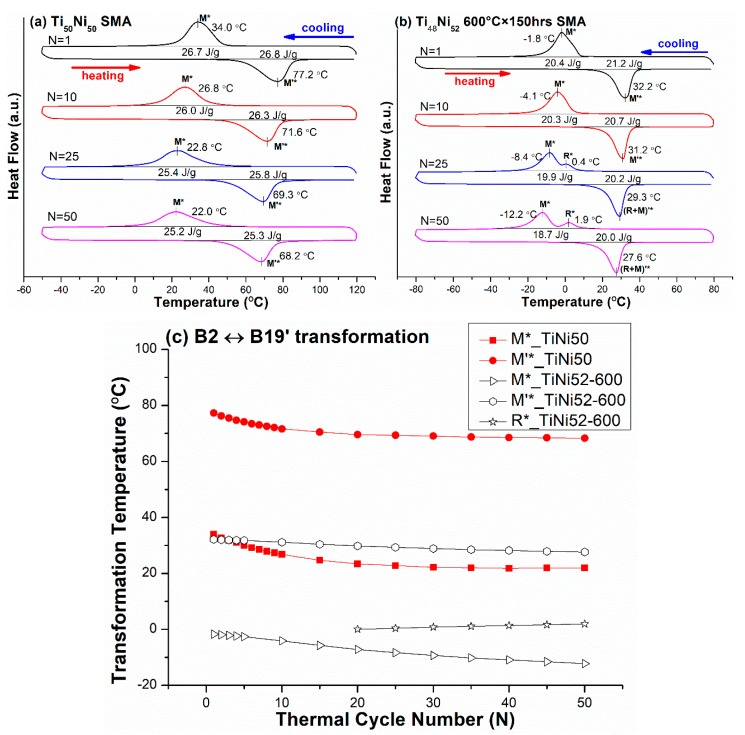
(**a**,**b**) The differential scanning calorimetry (DSC) curves of M* and M’* peak temperatures of the forward and reverse B2 ↔ B19′ martensitic transformations for Ti_50_Ni_50_ (TiNi50) shape memory alloy (SMA) and Ti_48_Ni_52_ aged at 600 °C × 150 h (TiNi52-600) SMA, respectively. (**c**) From (a) and (b), the variation of the transformation peak temperatures versus N is plotted for these two SMAs.

The effects of thermal cycling on transformation temperatures of as solution-treated specimens of Ti_50_Ni_50_ (abbreviated as TiNi50) and Ti_48_Ni_52_ SMAs were investigated. In this investigation, Ti_48_Ni_52_ specimens were further aged at 600 °C for 150 h (abbreviated as TiNi52-600) to form Ti_2_Ni_3_ precipitates and make the matrix exhibit B2 ↔ B19′ one-stage transformation [24]. Figure 1a,b shows the DSC curves of M* and M’* peak temperatures of the forward and reverse B2 ↔ B19′ martensitic transformations of TiNi50 and TiNi52-600 SMAs, respectively, thermal-cycled for N = 1, 10, 25, and 50 cycles. The T_max_ and T_min_ were set at 120 °C and −50 °C for TiNi50 SMA, and at 80 °C and −80 °C for TiNi50-600 SMA. From DSC, the transformation peak temperatures versus N for these two SMAs are plotted in Figure 1c. As shown in Figure 1, the TiNi50 SMA had only one M* peak of B2 → B19′ transformation during cooling and one M’* peak of B19′ → B2 transformation during heating for N = 1–50. TiNi52-600 SMA had the same transformation sequence as TiNi50 SMA for N = 1–20, but its forward transformation changed to B2 → R-phase → B19′ and the reverse transformation remained the same as that of B19′ → B2 for N = 20–50. This kind of transformation behavior has also been observed in Ti_49.8_Ni_50.2_ SMA, with the change of the transformation sequence at around N = 25 [18]. From Figure 1, it can be seen that, for TiNi50 SMA, the temperature difference between the transformation start and finish temperatures increased with increasing N, as also observed in other study [16]. For TiNi52-600 SMA, one can see that the thermal cycling introduced the R-phase in the forward transformation. This characteristic arises from the fact that the M* temperature of TiNi52-600 SMA was quite low, i.e., −1.8 °C for N = 1 and −12.2 °C for N = 50, as shown in Figure 1b, but that of TiNi50 SMA was not so low, i.e., 34.0 °C for N = 1 and 22.0 °C for N = 50, as shown in Figure 1a. As the M* (B2 → B19′) transformation competed with the R* (B2 → R-phase) transformation, the quite low M* temperature exhibited in the SMA, along with its M* temperature being further decreased by N, made the transformation free energy of B2 → B19′ higher than that of B2 → R-phase and thus induced the R-phase to form B2 → R-phase → B19′ during cooling. However, the temperature difference between M* and R* was not large enough, so the reverse transformation only exhibited thermodynamic B19′ → B2 transformation [17,18].

### 3.2. B2 ↔ R-Phase and B2 ↔ B19 One-Stage Transformation Sequences

**Figure 2:**
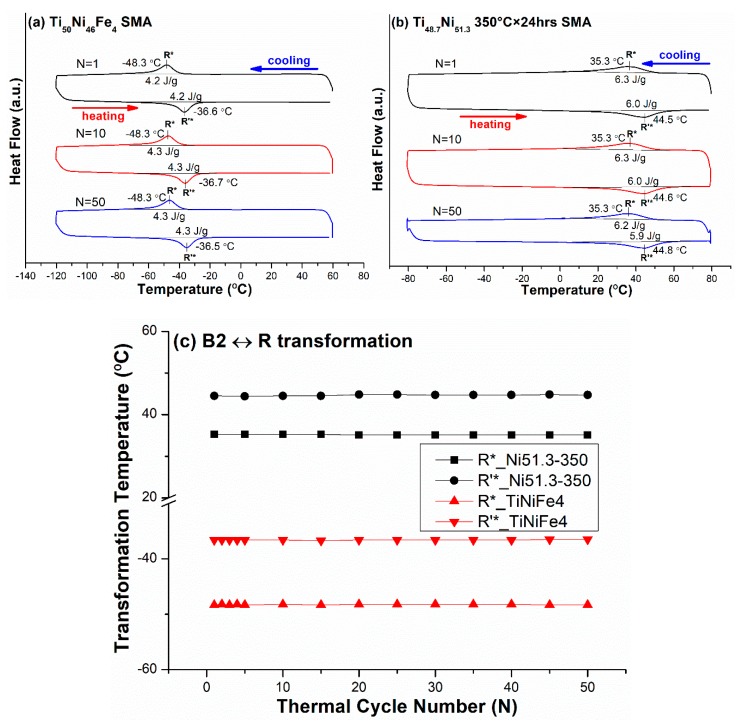
(**a**,**b**) The DSC curves of R* and R’* peak temperatures of the forward and reverse B2 ↔ R-phase premartensitic transformation for Ti_50_Ni_46_Fe_4_ (TiNiFe4) and Ti_48.7_Ni_51.3_ aged at 350 °C × 24 h (TiNi51.3-350) SMAs, respectively. (**c**) From (a) and (b), the curves of the transformation peak temperatures versus N are plotted for these two SMAs.

The effects of thermal cycling on the transformation temperatures of TiNi-based SMAs with different compositions but exhibiting the same B2 ↔ R-phase or B2 ↔ B19 one-stage transformation were also investigated. For the B2 ↔ R-phase transformation, as solution-treated Ti_50_Ni_46_Fe_4_ (abbreviated as TiNiFe4) and Ti_48.7_Ni_51.3_ SMAs were selected, with the latter being further aged at 350 °C for 24 h (abbreviated as TiNi51.3-350) [25]. For the B2 ↔ B19 transformation, as solution-treated Ti_50_Ni_35_Cu_15_ (abbreviated as TiNiCu15) and Ti_50_Ni_37_Pd_13_ (abbreviated as TiNiPd13) SMAs [7,26] were selected. Figure 2a,b shows the DSC curves of R* and R’* peak temperatures of the forward and reverse B2 ↔ R-phase premartensitic transformation of TiNiFe4 and TiNi51.3-350 specimens, respectively, thermal-cycled for N = 1, 10, and 50 cycles. The T_max_ and T_min_ were 60 °C and −120 °C for TiNiFe4 SMA, and 80 °C and −80 °C for TiNi51.3-350 SMA. From the DSC results, the transformation temperatures R* and R’ * versus N for both SMAs are plotted in Figure 2c. From Figure 2, it can be seen that, for both SMAs, the variation of R* and R’ * peak temperatures from N = 1 to N = 50 was less than 0.3 °C, and the hysteresis from N = 1 to N = 50 had almost no change. These characteristics of the thermal cycling effect exhibited in B2 ↔ R-phase transformation were quite different from those in the B2 ↔ B19′ counterpart shown in Figure 1.

**Figure 3:**
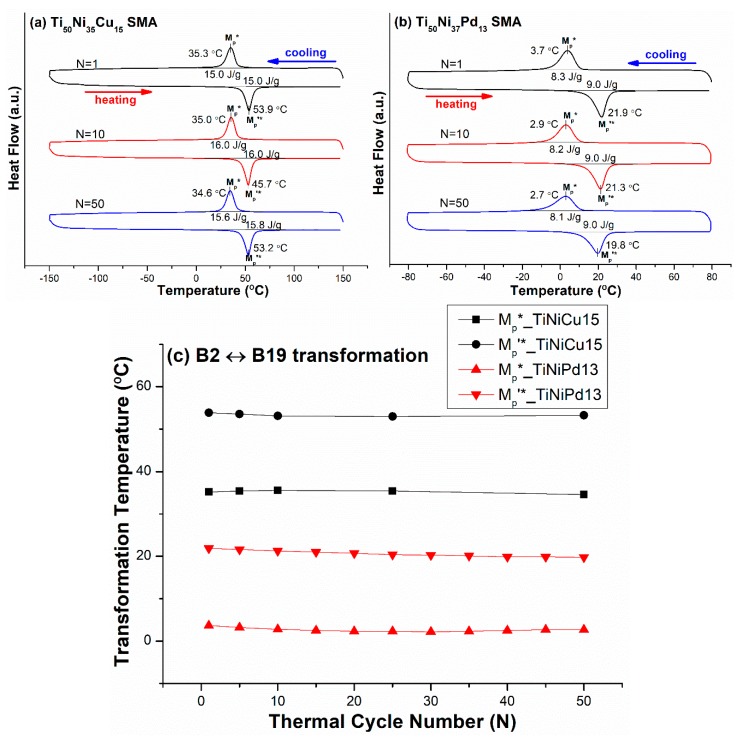
(**a**,**b**) The DSC curves of M_p_* and M_p_’* peak temperatures of the forward and reverse B2 ↔ B19 premartensitic transformation for Ti_50_Ni_35_Cu_15_ (TiNiCu15) and Ti_50_Ni_37_Pd_13_ (TiNiPd13) SMAs, respectively. (**c**) From (a) and (b), the curves of the transformation peak temperatures versus N are plotted for these two SMAs.

Figure 3a,b shows the DSC curves of the M_p_* and M_p_’* peak temperatures of the forward and reverse B2 ↔ B19 premartensitic transformation of the TiNiCu15 and TiNiPd13 specimens, respectively, thermal-cycled for N = 1, 10, and 50 cycles. The T_max_ and T_min_ were 150 °C and −150 °C for the TiNiCu15 SMA, and 80 °C and −80 °C for the TiNiPd13 SMA. Figure 3c indicates the variation of the transformation temperatures M_p_* and M_p_’* versus N from the DSC results shown in Figure 3a,b. As shown in Figure 3, the temperature decreases of M_p_* and M_p_’* were less than 1–2 °C for both SMAs, but were a little larger than those of R* and R’*, as shown in Figure 2. From Figure 3, it can also be seen that the temperature difference between the transformation start and finish temperatures of B2 ↔ B19 transformation from N = 1 to N = 50 also remained almost unchanged, like that of its B2 ↔ R counterpart shown in Figure 2.

### 3.3. B2 ↔ R-Phase ↔ B19′ Two-Stage Transformation Sequence

**Figure 4:**
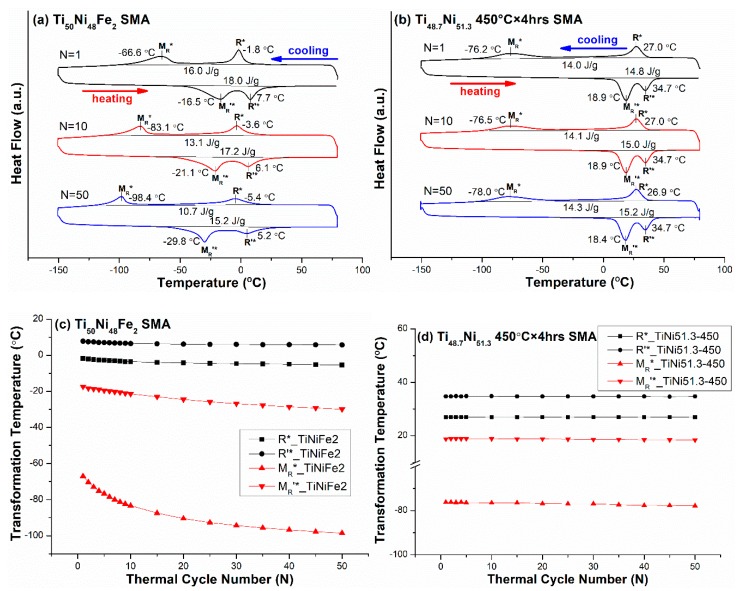
(**a**,**b**) The DSC curves of R*, M_R_*, M_R_’*, and R’* peak temperatures of the forward and reverse B2 ↔ R-phase ↔ B19′ two-stage martensitic transformation for Ti_50_Ni_48_Fe_2_ (TiNiFe2) and Ti_48.7_Ni_51.3_ aged at 450 °C × 4 h (TiNi51.3-450) SMAs, respectively. (**c**,**d**) The curves of the transformation peak temperatures versus N for TiNiFe2 and TiNi51.3-450 SMAs, respectively, in which the data come from (a,b).

The effects of thermal cycling on the transformation temperatures of TiNi-based SMAs with different compositions but exhibiting the same B2 ↔ R-phase ↔ B19′ two-stage martensitic transformation were also investigated. The as solution-treated Ti_50_Ni_48_Fe_2_ (abbreviated as TiNiFe2) and TiNi51.3 SMAs were selected, with the latter being further aged at 450 °C for 4 h (abbreviated as TiNi51.3-450). Figure 4a,b shows the DSC curves of the R*, M_R_* and M_R_’*, R’* peak temperatures of the forward and reverse B2 ↔ R-phase ↔ B19′ martensitic transformations of TiNiFe2 and TiNi51.3-450 SMAs, respectively. The T_max_ and T_min_ for both SMAs were 80 °C and −150 °C. From the DSC results, the variations of transformation temperatures, R*, M_R_*, M_R_’*, and R’* versus N for TiNiFe2 and TiNi51.3-450 SMAs are plotted in Figure 4c,d, respectively. As shown in Figure 4a,b, for N = 1, the hysteresis between M_R_* and M_R_’* and that between R* and R’* for TiNiFe2 SMA were 50.1 °C and 9.5 °C, respectively, and those for TiNi51.3-450 SMA were 95.1 °C and 7.7 °C, respectively. It is clear that the Ti_3_Ni_4_ ppts formed in TiNi51.3-450 SMA [1,2], but not in TiNiFe2 SMA, significantly suppressing the formation of B19′ martensite and thus widening the transformation hysteresis of M_R_* and M_R_’*. However, the coherent stress around the Ti_3_Ni_4_ ppts enhanced the formation of premartensitic R-phase and thus reduced the transformation hysteresis of R* and R’* [25]. As also shown in Figure 4, the M_R_* and M_R_’* peak temperatures for TiNiFe2 SMA from N = 1 to N = 50 were decreased by 31.8 °C and 13.3 °C, respectively, and those for TiNi51.3-450 SMA by 1.8 °C and 0.5 °C, respectively. The R* and R’* peak temperatures for TiNiFe2 SMA from N = 1 to N = 50 were decreased by 3.6 °C and 2.5 °C, respectively, and those for TiNi51.3-450 SMA, 0.1 °C and zero, respectively.

### 3.4. B2 ↔ B19 ↔B19′ Two-Stage Transformation Sequence

**Figure 5:**
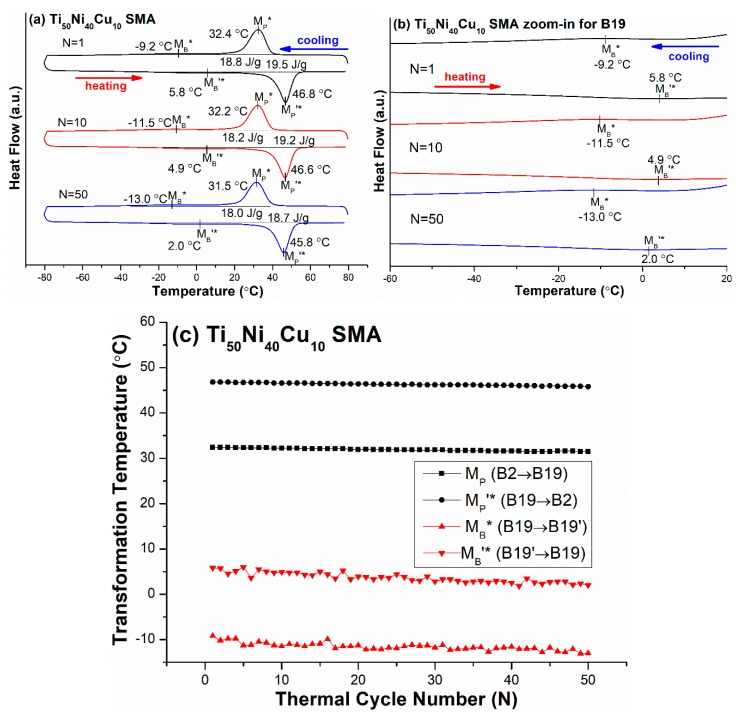
(**a**) The DSC curves of M_P_*, M_B_*, M_B_’*, and M_P_’* peak temperatures of the forward and reverse B2 ↔ B19 ↔ B19′ two-stage transformation for Ti_50_Ni_40_Cu_10_ (TiNiCu10) SMA; (**b**) the zoomed-in scale from (a) to clearly show the M_B_*, M_B_’* peaks. (**c**) From (a), the curves of the transformation peak temperatures versus N are plotted.

The Ti_50_Ni_40_Cu_10_ (abbreviated as TiNiCu10) SMA was selected to study the effects of thermal cycling on the transformation temperatures of TiNi-based SMAs exhibiting B2 ↔ B19 ↔B19′ two-stage martensitic transformation [7]. Figure 5a shows the DSC curves of the M_P_*, M_B_*, M_B_’*, and M_P_’* peak temperatures of the forward and reverse B2 ↔ B19 ↔B19′ martensitic transformations, and Figure 5b is the zoomed-in scale from Figure 5a to clearly show the M_B_* and M_B_’* peaks. The T_max_ and T_min_ were 80 °C and −80 °C, respectively. From the DSC results, the transformation temperatures M_P_*, M_B_*, M_B_’*, and M_P_’* versus N are plotted in Figure 5c. As shown in Figure 5, the M_B_* and M_B_’* peak temperatures of B19 ↔B19′ transformation from N = 1 to N = 50 were both decreased by 3.8 °C, which is much lower than those of the R ↔ B19′ and B2 ↔ B19′ transformations shown in Figure 4c and Figure 1c, respectively. As also shown in Figure 5, the M_P_* and M_P_’* peak temperatures of B2 ↔ B19 premartensitic transformation from N = 1 to N = 50 were decreased by 0.9 °C and 1.0 °C, respectively, which was a little larger than that of B2 ↔ R shown in Figure 2. At the same time, the temperature difference of the transformation start and finish temperatures for N = 1 to N = 50 had no obvious changes, as indicated in Figure 5a.

## 4. Discussion

Table 1 displays the nomenclature for the different SMAs and various transformation sequences used in this study. Miyazaki et al. revealed that the decrease of the transformation temperature of TiNi-based SMAs during thermal cycling results from the introduction of dislocations [16]. We propose two factors that affect the ease of introducing dislocations during thermal cycling. One is the SMA’s intrinsic hardness, i.e., the alloy’s yielding stress. The other is the shear strain, ***s***, which is associated with the martensitic transformation exhibited in TiNi-based SMAs. It is well-known that, with a larger yielding stress, fewer dislocations are induced during SME/PE application, thus leading to better SMA shape memory properties [27]. This characteristic implies that SMA processes with higher yielding stress can introduce fewer dislocations during thermal cycling, and thus, the suppression of the martensitic transformation temperature will be reduced more. Table 2 lists the hardnesses at RT of the TiNi-based SMAs used in this study. Table 3 lists the reported shear strains, ***s***, of the different martensitic transformations exhibited in TiNi-based SMAs.

### 4.1. Effect of the Hardness of the SMA

Table 2 shows that the hardnesses of TiNiFe2 and TiNi51.3-450 SMAs at RT were 194 HV and 335 HV, respectively, in which the TiNiFe2 SMA was in the B2 parent phase at RT, but the TiNi51.3-450 SMA possessed two phases (B2 + R) at RT, as revealed in Figure 4a,b, respectively. Because the hardness of the R-phase is lower than that of the B2 phase [33], the intrinsic hardness of the TiNi51.3-450 SMA in the B2 phase should be higher than 335 HV due to the occurrence of Ti_3_Ni_4_ precipitation hardening. Compared with the results shown in Figure 4a,b for the same B2 ↔ R-phase ↔ B19′ transformation sequence, one can find that when the hardness of the SMA was higher, the thermal cycling effect was smaller. In addition, Figure 4a,b also demonstrates that the temperature difference, i.e., the transformation hysteresis, of the transformation peak temperatures of (R* and R’*) and (M_R_* and M_R_’*) from N = 1 to N = 50 significantly increased for TiNiFe2 SMA but had almost no change for TiNi51.3-450 SMA. This phenomenon indicates that the hardness of the SMA also affected the change of the transformation hysteresis by thermal cycling.

### 4.2. Effect of Shear Strain, s, Associated with Martensitic Transformation

From Figure 1, Figure 2, Figure 3, Figure 4 and Figure 5, the magnitudes of the decrease of temperature for the forward transformation of each transformation sequence during thermal cycling from N = 1 to N = 50 are listed in Table 4. From Table 4, it can be seen that, for each transformation sequence in one-stage transformation or in two-stage transformation, a higher ***s*** value associated with the martensitic transformation led to a larger decrease of the transformation temperature by thermal cycling. This phenomenon arises from the fact that a higher ***s*** value will induce larger shear strain during transformation, and thus introduce more dislocations during thermal cycling in alloys. Comparing Figure 1 with Figure 4, it can be seen that both TiNi50 and TiNiFe2 SMAs had significant thermal cycling effects on the decrease of M* (B2 → B19′) and M_R_* (R-phase → B19′) temperatures, respectively, due to both SMAs having low hardnesses and high ***s*** values associated with martensitic transformations. Additionally, from Table 4 and Figure 2, it can be seen that the thermal cycling effect on the decrease of the R* (B2 → R-phase) temperature for both TiNiFe4 and TiNi50.3-350 SMAs was zero from N = 1 to N = 50, due to the R* transformation having quite a low ***s*** value, such as the value of 0.0265 listed in Table 3. However, for the B2 → R-phase → B19′ transformation sequence exhibited in TiNiFe2 and TiNi51.3-450 SMAs, their R* and M_R_* temperatures decreased by 3.6 °C and 31.8 °C for the former SMA, and by zero and 1.8 °C for the latter SMA, respectively, from N = 1 to N = 50. As seen in Table 2, the hardness of TiNiFe2 SMA was much lower than that of TiNi5.13-450 SMA. This characteristic indicates that the intrinsic hardness of the SMA seemed to have a more significant effect than the ***s*** value on the decrease of transformation temperature by thermal cycling. In addition, from Table 3, it can be seen that the M_R_* transformation was associated with a higher ***s*** value than the R* transformation, and thus had a higher thermal cycling effect to introduce more dislocations. These introduced dislocations will directly affect the R* transformation temperature during the next thermal cycle N for SMAs exhibiting B2 → R-phase → B19′ two-stage transformation. This fact causes the suppression of the R* transformation temperature by thermal cycling to be more significant in TiNiFe2 SMA than in TiNiFe4 SMA, due to the latter only exhibiting B2 → R-phase one-stage transformation instead of B2 → R-phase → B19′ two-stage transformation.

From Table 3 and Table 4, it is clear that the ***s*** value associated with B2 → B19 transformation was higher than that associated with B2 → R-phase transformation; thus, the decrease of the temperature of M_P_* (B2 → B19) transformation exhibited in TiNiCu15 and TiNiPd13 SMAs due to thermal cycling was larger than that of the R* transformation exhibited in B2 ↔ R-phase one-stage transformation, such as in TiNiFe4 and TiNi51.3-350 SMAs. Figure 5 indicates that TiNiCu10 SMA also underwent two-stage transformation, but it was B2 ↔ B19 ↔ B19′, instead of the B2 ↔ R-phase ↔ B19′ transformation exhibited in TiNiFe2 SMA. Comparing these two-stage transformations, Table 4 shows that the decrease of transformation temperatures affected by the thermal cycling was much lower in TiNiCu10 SMA than in TiNiFe2 SMA. This phenomenon arises from the fact that the ***s*** value of the B19 → B19′ transformation is lower than that of its R-phase → B19′ counterpart, as indicated in Table 4; because the s value of the B2→R transformation is significantly lower than that of its B2→B19 counterpart, thus, fewer dislocations will be introduced by thermal cycling to directly affect the B2 → B19 transformation in the next thermal cycle N, although the exact ***s*** values associated with the B19 → B19′ and R-phase → B19′ transformations have not been reported yet.

### 4.3. Characteristics of B2 → R-Phase Transformation Temperature Affected by Thermal Cycling in B2 → R and B2 → R-phase → B19′ Transformations

Carefully examining Table 4, it can be seen that the R*(B2 → R-phase) transformation appeared in the one-stage transformation of the TiNiFe4 and TiNi51.3-350 SMAs, and also in the B2 → R-phase → B19′ two-stage transformation of the TiNi52-600, TiNiFe2, and TiNi51.3-450 SMAs. Among these SMAs, the R* transformation temperature exhibited in TiNi52-600 SMA increased slightly with N from 20 to 50; i.e., the R* transformation temperature was not suppressed during thermal cycling as it started to appear. This characteristic is uncommon, and has also been observed in thermal-cycled Ti_49.8_Ni_50.2_ SMA with N from 25 to 50 [18]. However, as mentioned in the previous section, the R* temperatures exhibited in TiNiFe4, TiNi51.3-350, and TiNi51.3-450 SMAs remained unchanged, and that in TiNiFe2 SMA was suppressed with increasing N because the R* transformation of the former three SMAs was associated with small ***s*** values and/or high intrinsic hardness, but that of the latter TiNiFe2 SMA was directly affected by its sequential R → B19′ transformation exhibited in a two-stage transformation and its low intrinsic hardness. Miyazaki et al. proposed that dislocations were introduced in thermal-cycled TiNi SMAs [16]. We propose that the slight increase in the R* temperature with increasing N in TiNi52-600 SMA may result from enhancement of the formation of R-phase by the strain field around these thermal-cycled dislocations. However, more study is needed to confirm this.

## 5. Conclusions

In this study, the thermal cycling effects on the transformation temperatures of different transformation sequences exhibited in TiNi-based SMAs, including B2 ↔ B19′, B2 ↔ R-phase, B2 ↔ R-phase ↔ B19′, B2 ↔ B19, and B2 ↔ B19 ↔ B19′ transformations, were investigated. Experimental results indicate that the intrinsic hardness and the shear strain, ***s***, associated with martensitic transformation, of the SMAs are two important factors that affect the decrease of transformation temperatures by thermal cycling, because these two factors are related to the ease of introducing dislocations during thermal cycling. For the one-stage transformation sequence, the degree of the temperature decrease by thermal cycling were in the order of B2 ↔ B19′ > B2 ↔ B19 > B2 ↔ R-phase, according to the magnitude order of their ***s*** values. For the same reason, the degree of temperature decrease of the R-phase ↔ B19′ transformation by thermal cycling in the B2 ↔ R-phase ↔ B19′ two-stage transformation of TiNiFe2 SMA was larger than that of the B19 ↔ B19′ transformation in the B2 ↔ B19 ↔ B19′ two-stage transformation of TiNiCu10 SMA. Both TiNiFe2 and TiNi51.3-450 SMAs exhibited the same B2 ↔ R-phase ↔ B19′ transformation sequence, but the latter hadmuch higher hardness than the former due to the enhancement of the precipitation hardening by Ti_3_Ni_4_ ppts. This characteristic suppressed the R-phase ↔ B19′ transformation temperature much more in the TiNiFe2 SMA than in TiNi51.3-450 SMA. In addition, the thermal-cycled dislocations induced by the R-phase ↔ B19′ transformation in TiNiFe2 SMA could directly affect the sequential B2 ↔ R-phase transformation in the next thermal cycle and caused obvious decrease of the B2 ↔ R-phase transformation temperature. The TiNi52 SMA aged at 600 °C for 100 h underwent B2 ↔ B19′ one-stage transformation, but as the thermal cycling number increased from 20 to 50, it changed to B2 ↔ R-phase ↔ B19′ two-stage transformation, and the B2 ↔ B19′/R-phase ↔ B19′transformation temperature was decreased but its B2 ↔ R-phase counterpart was raised slightly by thermal cycling. The increase in the transformation temperature by thermal cycling is uncommon, and this characteristic may have arisen from the strain field induced by thermal-cycled dislocations favoring the formation of R-phase.

## Figures and Tables

**Table 1:** The nomenclature for different SMAs and various transformation sequences used in this study.

SMAs (in at. %)	DSC Tests for Thermal Cycling at T_min_/T_max_ (°C)	Transformation Sequence	Forward Transformation Peak Temperature at N = 1 (°C)
Ti_50_Ni_50_ (TiNi50)	−50/120	B2 ↔ B19′	34.0
Ti_48_Ni_52_ 600 °C × 150 h (TiNi52-600)	−80/80	B2 ↔ B19′ (N = 1–20) B2 → R → B19′ (N = 21–50) B19′ → B2 (N = 21–50)	−1.8
Ti_50_Ni_46_Fe_4_ (TiNiFe4)	−120/60	B2 ↔ R	−48.3
TiNi_51.3_ 350 °C × 24 h (TiNi51.3-350)	−80/80	B2 ↔ R	35.3
Ti_50_Ni_35_Cu_15_ (TiNiCu15)	−150/150	B2 ↔ B19	35.3
Ti_50_Ni_37_Pd_13_ (TiNiPd13)	−80/80	B2 ↔ B19	3.7
Ti_50_Ni_48_Fe_2_ (TiNiFe2)	−150/80	B2 ↔ R ↔ B19′	R*: −1.8, M*: −66.6
TiNi_51.3_ 450 °C × 4 h (TiNi51.3-450)	−150/80	B2 ↔ R ↔ B19′	R*: 27.0, M*: −76.2
Ti_50_Ni_40_Cu_10_ (TiNiCu10)	−80/80	B2 ↔ B19 ↔ B19′	M_P_*: 32.4, M_B_*: −9.2

**Table 2:** Hardness at room temperature of TiNi-based SMAs used in this study.

SMAs (in at. %)	Hardness (Hv)	Phase(s) of Matrix at Room Temperature
Ti_50_Ni_50_ (TiNi50)	174 ± 6	B2 and B19′ (with B19′ being the major phase)
Ti_48_Ni_52_ 600 °C × 150 h (TiNi52-600)	228 ± 7	B2 and Ti_2_Ni_3_ ppts
Ti_50_Ni_46_Fe_4_ (TiNiFe4)	196 ± 5	only B2
TiNi_51.3_ 350 °C × 24 h (TiNi51.3-350)	387 ± 8	(B2 + R) and Ti_3_Ni_4_ ppts (with R being the major phase)
Ti_50_Ni_35_Cu_15_ (TiNiCu15)	220 ± 9	only B19
Ti_50_Ni_37_Pd_13_ (TiNiPd13)	177 ± 4	only B2
Ti_50_Ni_48_Fe_2_ (TiNiFe2)	194 ± 7	only B2
TiNi_51.3_ 450 °C × 4 h (TiNi51.3-450)	335 ± 6	(B2 + R) and Ti_3_Ni_4_ ppts (with R-phase being the major phase)
Ti_50_Ni_40_Cu_10_ (TiNiCu10)	177 ± 10	B2 and B19 (with B19 being the major phase)

**Table 3:** The associated shear strain, ***s***, of different martensitic transformations exhibited in TiNi-based SMAs.

Martensitic Transformation Type	The Associated Twinning Mode in Martensite	Magnitude of the Shear Strain, *s*
B2 ↔ B19′ martensitic transformation	<011 >_M_ type II	0.2804 [28]
	(001)_M_/(100)_M_ compound	0.2348 [28,29]
	{$\bar{1}\bar{1}1$}_M_ type I	0.30961 [28]
B2 ↔ R premartensitic transformation	{1121}_R_ i.e., {100}_B2_	0.0265 * [30,31]
	{$11\bar{2}\bar{2}$}_R_ i.e., {011}_B2_	0.0265 * [30,31]
B2 ↔ B19 premartensitic transformation	{111}_M_ type I	0.17 [32]
	{011}_M_ compound	0.11 [32]

* The distortion angle α of the premartensite R-phase is 89.56°.

**Table 4:** Magnitude of the temperature decrease for the forward transformation of TiNi-based SMAs during thermal cycling from N = 1 to N = 50. The ***s*** value for each transformation is taken from Table 3.

TiNi-Based SMAs	Transformation Type	*s* Value	The Decrease of the Forward Transformation Temperature from N = 1 to N = 50 (°C)
Ti_50_Ni_50_ (TiNi50)	B2 → B19′	0.2804	−12.0
Ti_48_Ni_52_ 600 °C × 150 h (TiNi52-600)	B2 → B19′	0.2804	−6.6 ^§^
	B2 → R	0.0265	+1.9 ^§§^
	R → B19′	<0.2804 *^,†^	−3.8 ^§§^
Ti_50_Ni_46_Fe_4_ (TiNiFe4)	B2 → R	0.0265	0
TiNi_51.3_ 350 °C × 24h (TiNi51.3-350)	B2 → R	0.0265	0
Ti_50_Ni_35_Cu_15_ (TiNiCu15)	B2 → B19 {011}_M_ compd. twin + {111}_M_ type I twin	0.11~0.17	−0.7
Ti_50_Ni_37_Pd_13_ (TiNiPd13)	B2 → B19 {111}_M_ type I twin	0.17	−1.0
Ti_50_Ni_48_Fe_2_ (TiNiFe2)	B2 → R	0.0265	−3.6
	R → B19′	<0.2804 *^,†^	−31.8
TiNi_51.3_ 450 °C × 4 h (TiNi51.3-450)	B2 → R	0.0265	0
	R → B19′	<0.2804 *^,†^	−1.8
Ti_50_Ni_40_Cu_10_ (TiNiCu10)	B2 → B19	0.11~0.17	−0.9
	B19 → B19′	<0.2804 *^,†^	−3.8

* No reported. ^†^ The total ***s*** value of B2 → B19′ is 0.2804, therefore, for B2 → R-phase → B19′ and B2 → B19 → B19′ two-stage transformations, the ***s*** value of B19 → B19′ should be less than that of R-phase → B19′ because the ***s*** value of B2 → R-phase is much lower than that of B2 → B19. ^§^ From N = 1 to N = 20. ^§§^ From N = 20 to N = 50.
